# DNA methylation analysis at distal and proximal promoter regions of the oestrogen receptor gene in breast cancers

**DOI:** 10.1038/sj.bjc.6690631

**Published:** 1999-08

**Authors:** H Iwase, Y Omoto, H Iwata, T Toyama, Y Hara, Y Ando, Y Ito, Y Fujii, S Kobayashi

**Affiliations:** Department of Surgery II, Nagoya City University Medical School, Kawasumi 1, Mizuho-ku, Nagoya, 467-8601, Japan

**Keywords:** oestrogen receptor α, DNA methylation, hormone resistance, breast cancer

## Abstract

Oestrogen receptor α (ER-α) gene has two specific promoters, distal (P0) and proximal (P1), which induce almost identical transcripts in size due to different splicing. We examined the methylation at both promoter regions of the ER-α gene using *Hpa*II, a methylation-sensitive restriction enzyme, prior to polymerase chain reaction (PCR) amplification. To confirm the results of PCR-based methylation analysis, Southern hybridization was also performed. Twenty of 29 patients with ER-α-positive tumours and five of 27 with ER-α-negative tumours were unmethylated at the P1 promoter region of the ER-α gene. The incidence of methylation was highly negatively correlated with ER-α expression (*P* = 0.0002). A similarly negative correlation was observed at the P0 promoter region of the ER-α gene (*P* = 0.0154). Additionally, the tumours with the ER-α gene hypermethylated at both promoter regions had definitely negative ER-α values. It was suggested that this epigenetic change might control ER-α expression, and might play an important role in the loss of hormone-dependence in breast cancer. © 1999 Cancer Research Campaign


*British Journal of Cancer* (1999) **80**(12), 1982–1986
© 1999 Cancer Research Campaign
Article no. bjoc.1999.0631

### DNA methylation analysis at distal and proximal promoter regions of the oestrogen receptor gene in breast cancers

**H Iwase, Y Omoto, H Iwata, T Toyama, Y Hara, Y Ando, Y Ito, Y Fujii and S Kobayashi**

Department of Surgery II, Nagoya City University Medical School, Kawasumi 1, Mizuho-ku, Nagoya 467-8601, Japan

**Summary** Oestrogen receptor α (ER-α) gene has two specific promoters, distal (P0) and proximal (P1), which induce almost identical transcripts in size due to different splicing. We examined the methylation at both promoter regions of the ER-α gene using *Hpa*II, a methylation-sensitive restriction enzyme, prior to polymerase chain reaction (PCR) amplification. To confirm the results of PCR-based methylation analysis, Southern hybridization was also performed. Twenty of 29 patients with ER-α-positive tumours and five of 27 with ER-α-negative tumours were unmethylated at the P1 promoter region of the ER-α gene. The incidence of methylation was highly negatively correlated with ER-α expression ($P = 0.0002$). A similarly negative correlation was observed at the P0 promoter region of the ER-α gene ($P = 0.0154$). Additionally, the tumours with the ER-α gene hypermethylated at both promoter regions had definitely negative ER-α values. It was suggested that this epigenetic change might control ER-α expression, and might play an important role in the loss of hormone-dependence in breast cancer.

**Keywords:** oestrogen receptor α; DNA methylation; hormone resistance; breast cancer

Human breast cancer is a typical hormone-dependent tumour, and various endocrine treatments have been employed in advanced or recurrent cases. These treatments have also been performed as a part of post-operative adjuvant therapy. The measurement of oestrogen receptor α (ER-α) in cancer tissues is now an important procedure in order to discriminate between hormone-dependent and -independent tumours. Although about 60% of patients with ER-α in their cancer tissues responded to endocrine therapies, fewer than 10% of patients without ER-α also responded (McGuire et al, 1991). Furthermore, ER-α-negative tumours are associated with poorer histological differentiation, higher growth fraction and a somewhat poorer clinical outcome (McGuire et al, 1991). Hormone resistance could partly result from the loss of the ER-α protein, or might be due to the presence of mutant/variant ER-α in breast cancer (McGuire et al, 1991; Katzenellenbogen et al, 1997). However, no significant alterations such as insertions, deletions, rearrangements, or point mutations within the ER-α gene have been reported (Karnik et al, 1994; Roodi et al, 1995). Thus, genetic alterations of the ER-α gene at the DNA level might account for only a portion of ER-α expression.

DNA methylation is known to be involved in eukaryotic gene control, and can effect development and tumorigenesis (Falette et al, 1990). The ER-α gene was found to be methylated in placental tissues, but normal breast tissues exhibit a different methylation pattern, as assessed by *Hpa*II and *Msp*I restriction enzyme-digests (Falette et al, 1990). In addition, specific sites in the hormone-binding domain of the ER-α gene were observed to be differently methylated in different human breast tumour specimens (Falette et al, 1990). In particular, previous studies correlated the lack of ER-α gene expression in ER-α-negative breast tumour cells with hypermethylation of a CpG island in the 5′ region of the ER-α gene (Ottaviano et al, 1994; Ferguson et al, 1995). Thus, DNA methylation may be an additional molecular measure of the genetic heterogeneity in breast cancer.

ER-α has two specific promoters, distal (P0) and proximal (P1), which induce almost identical transcripts in size due to different splicing, and the only difference between the two transcripts is the most 5′ untranslated 164 and 120 bases, which are unique for each transcript (Grandien et al, 1995). Hayashi et al (1997) reported that the enhancement of the ER-α mRNA expression from the distal promoter played an essential role in the mechanisms of over-expressing ER-α protein in human mammary tumours, implying that a tumour-specific regulation of ER-α expression involved use of the distal promoter. However, Grandien et al (1995) reported that both promoters were active in MCF-7 cancer cells, and that only the P1 promoter was transcribed in ZR-75-1 breast cancer cells.

In this paper we examined alterations in DNA methylation at the distal and proximal promoter regions of the ER-α gene using polymerase chain reaction (PCR)-based methylation assay and Southern blot assay in breast cancers. We also discuss the clinical significance of this epigenetic change.

### MATERIALS AND METHODS

### Patients and DNA extraction

Tissues from 56 patients with primary breast cancers were obtained by surgical resection in the Second Department of Surgery of Nagoya City University Medical School. None of the patients had a familial history of breast cancer. Of the 56 tumours,

*Received 26 August 1998*
*Revised 22 January 1999*
*Accepted 11 March 1999*

*Correspondence to:* H Iwase

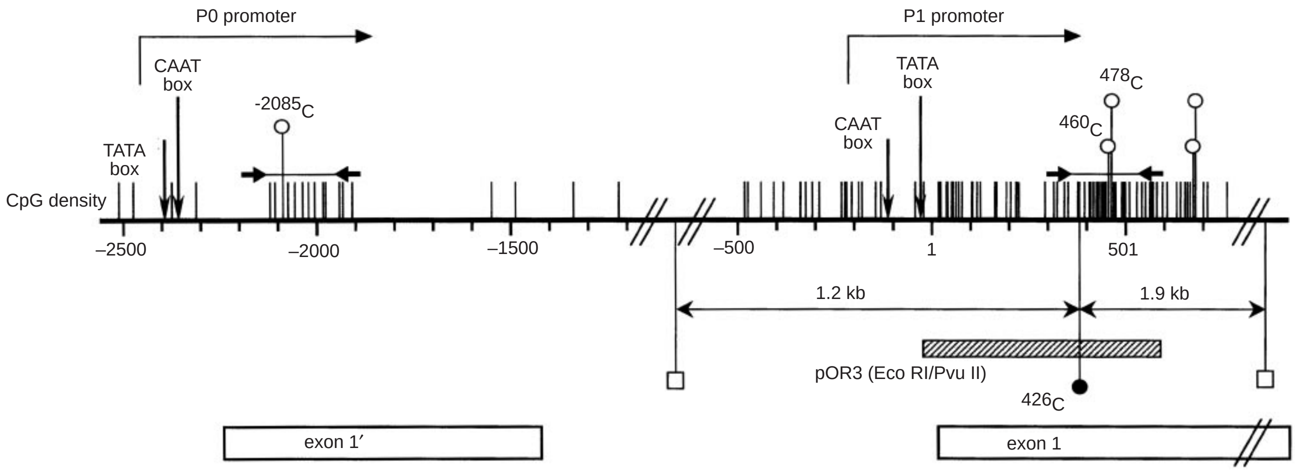

**Figure 1** CpG density and restriction sites of the ER gene and its upstream region. The ER-α has two specific promoters, distal (P0) and proximal (P1), that are almost identical in size due to different splicing. Methylation was examined using PCR-based assay. PCR product (292 bp) at the P0 promoter region included one CCGG site (open circle, $^{-2085}$C), and that (294 bp) at the P1 promoter region included two CCGG sites ($^{460}$C and $^{478}$C). *Not*I site (closed circle, $^{426}$C) is near the CCGG site at the P1 promoter region. Arrows show the primers for PCRs

20 were papillo-tubular carcinomas, eight were solid-tubular carcinomas, 25 were scirrhous carcinomas and three were invasive lobular carcinomas. Patient ages at the operation ranged from 33 to 86 years (median 52). Genomic DNA from the breast cancer specimens was extracted by standard techniques.

### Oestrogen and progesterone receptor determinations

Cytosolic ER-α and progesterone receptor (PR) levels were measured using enzyme immunoassay (ER– and PgR–EIA, Abbott Laboratories, Chicago, IL, USA). A positive ER-α and PR status was defined as more than 15 fmol mg$^{-1}$ protein.

### PCR-based methylation assay

We examined the methylation status at P0 and P1 promoter regions of the ER-α gene (Figure 1). A PCR-based assay was performed as described previously (Gonzalez-Zuluenta et al, 1995), with some modification. One microgram of genomic DNA was digested overnight with 10 units of the methylation-sensitive restriction enzyme *Hpa*II under conditions specified by the manufacturer (Takara, Kyoto, Japan). Fifty nanograms of the digested DNA were amplified by PCR. The primer sequences are 5′-TCT-CCCCTCACTCCCCACTGC-3′, 5′-GAAATCAAAACAAGCC-TACCC-3′ for the P0 promoter region, 5′-AGCAG-CAAGCCCGCCGTGTACAAC-3′ (368–391) and 5′-CTCGCG-CACCGTGTAGCCGCTGGG-3′ (638–661) for the P1 promoter region. Conditions were as follows: 95°C for 5 min, 25 cycles of 94°C for 1 min, annealing temperature (60°C for P0 and 58°C for P1) for 1 min and 70°C for 1 min, followed by incubation at 72°C for 5 min. PCR conditions were determined by cycle curve and DNA concentration curve. To rule out the possibility of false positives due to incomplete digestion and overcycling of the PCR amplifications, the digestions of each sample and PCR amplification were performed at least twice in independent experiments. Undigested DNA and *Msp*I digested DNA samples were amplified as positive and negative controls respectively. PCR products were resolved on 1.5% agarose gels. Loss or reduction of the PCR products following digestion by *Hpa*II was assessed as unmethylation.

### Southern hybridization

To confirm the results of PCR-based methylation analysis, Southern hybridization was performed. Ten micrograms of genetic DNA were digested with 100 units of *Not*I overnight, and subsequently digested with 100 units of *Eco*RI. After electrophoresis on a 1.2% agarose gel, samples were transferred to Hybond N$^+$ nylon membrane (Amersham Corp., Buckinghamshire, UK). Filters were hybridized with a pOR3 probe, which was generously provided by Professor P Chambon, and labelled with [$\alpha$-$^{32}$P]dCTP using a Multiprime-labelling kit (Amersham), as previously described.

### RESULTS

### DNA methylation at P0, P1 promoter regions of the ER-α gene in breast cancers

We investigated 56 breast cancers for methylation at the P0 and P1 promoter regions of the ER-α gene with a PCR-based assay. Unmethylation at the P0 and P1 promoter regions was observed in 15 (26.8%) and 25 (44.6%) of 56 breast cancers using PCR-based assay respectively (Figure 2A, B and Table 1). To confirm the results of the PCR-based methylation assay, we also performed genomic Southern hybridization. In unmethylated samples, double digestion with *Eco*RI and *Not*I yielded 1.9 kb and 1.2 kb fragments. If samples were methylated, 3.1 kb bands would be detected. We studied ten samples that showed abnormal methylation in PCR-based assay. As shown in Figure 2B, four breast cancers showed methylation of the *Not*I sites in Southern hybridization assay, and one breast cancer showed unmethylation. The agreement between the two methods was nine of ten (90%). On the other hand, frequency of unmethylation at the P1 promoter region was higher than that at P0 promoter region. The methylation at the P1 region correlated with that at the P0 region (Table 2). Additionally, the tumours with the ER-α gene hypermethylated at both promoter regions had a definitely negative ER-α protein value (Figure 3).

*© 1999 Cancer Research Campaign*

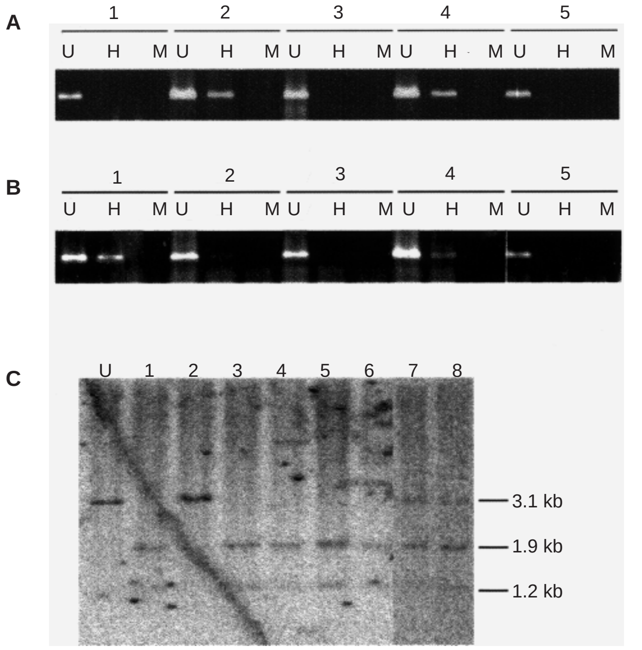

**Figure 2** Examples of methylation analysis. (**A**) Examples of PCR-based methylation assay at the P1 promoter region of the ER-α gene. U, undigested; H, digested by *Hpa*II; M, digested by *Msp*I. Cases 1, 3 and 5 are shown as methylation. Cases 2 and 4 are assessed as unmethylation. (**B**) Examples of PCR-based methylation assay at the P0 region of the ER-α gene. Cases 2, 3 and 5 are assessed as methylation. Cases 1 and 4 are assessed as unmethylation. (**C**) Southern blot analysis following digestion by *Not*I. U, undigested control. Cases 1, 3, 4, 5 and 6 can be assessed as unmethylation, and case 2 is assessed as methylation and cases 7 and 8 are assessed as hetero types with both methylation and unmethylation

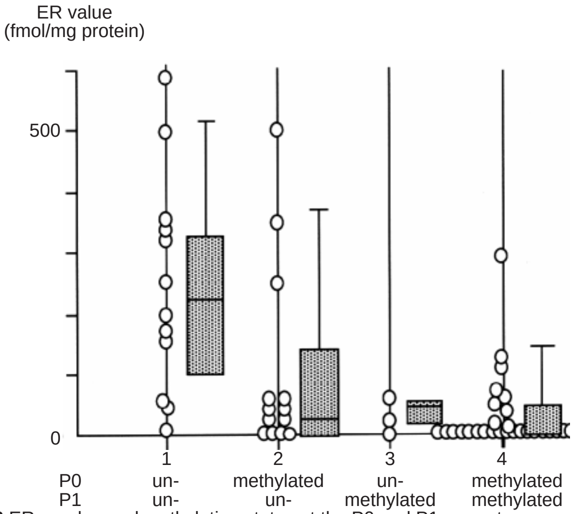

**Figure 3** ER-α value and methylation status at the P0 and P1 promoter regions of the ER gene. The boxes represent the mean and the 70% confidential interval; bars s.d. Group 1, unmethylation at both promoter regions, had higher ER-α values than other groups (1 vs 2; *P* = 0.0057, 1 vs 3; *P* = 0.0132, 1 vs 4; *P* < 0.001, statistical analysis by Fisher's protected least significant difference)

### Methylation status and clinicopathologic factors in breast cancers

Twenty of 29 patients with ER-α protein-positive tumours, and five of 27 with negative tumours were unmethylated at the P1 promoter region of the ER-α gene. The incidence of methylation was highly negatively correlated with ER-α expression ($P = 0.0002$). A similarly negative correlation was observed at the P0 promoter region ($P = 0.0154$). Methylation at the P0 and P1 regions was also negatively correlated with PR expression. However, there was no correlation between methylation and any other clinicopathologic factors (Table 1).

### DISCUSSION

Tumours failing to express ER-α would be oestrogen-independent and would most likely be resistant to anti-oestrogen therapy. Johnston et al, 1995) reported that the overall frequency of ER-α expression measured by immunohistochemical assay was reduced from 51% (37/72) at the initial operation to 29% (21/72) at progression or relapse. Thus, hormone resistance would partly result from the loss of the ER-α protein. Roodi et al (1995) reported that, in the majority of primary breast cancers, the ER α-negative phenotype was due to deficient ER-α expression at the

**Table 1** Relationship between DNA methylation at the P0 and P1 promoter regions of the oestrogen receptor gene and clinicopathologic factors

| | | P0 region | | | P1 region | | |
|---|---|---|---|---|---|---|---|
| | | Methylated | Unmethylated | | Methylated | Unmethylated | |
| ER | + | 17 | 12 | | 9 | 20 | |
| | – | 24 | 3 | *P* = 0.0154* | 22 | 5 | *P* = 0.0002** |
| PR | + | 16 | 8 | | 9 | 15 | |
| | – | 25 | 7 | *P* = 0.37 | 22 | 10 | *P* = 0.030* |
| Age | < 50 | 17 | 6 | | 15 | 8 | |
| | ≥ 50 | 24 | 9 | *P* > 0.999 | 16 | 17 | *P* = 0.2785 |
| n | + | 17 | 7 | | 13 | 11 | |
| | – | 24 | 8 | *P* = 0.7677 | 18 | 14 | *P* > 0.999 |
| t | < 2 cm | 5 | 3 | | 5 | 3 | |
| | ≥ 2 cm | 36 | 12 | *P* = 0.6676 | 26 | 22 | *P* = 0.7198 |
| HG | I | 10 | 6 | | 9 | 7 | |
| | II | 17 | 7 | *P* = 0.1757 | 13 | 11 | *P* = 0.9861 |
| | III | 7 | 0 | | 4 | 3 | |
| Total | | 41 | 15 | | | 31 | 25 |

ER: oestrogen receptor, PR: progesterone receptor, n: axillary lymph node metastasis, t: tumor size, HG: histological grade, *P*: Fisher's exact probability test.

 *© 1999 Cancer Research Campaign*

**Table 2** Relationship between methylation at the P0 and P1 promoter regions of the oestrogen receptor gene

| | P1 region | | | |
|---|---|---|---|---|
| | Methylated | Unmethylated | Total | |
| P0 region | | | | |
| Methylated | 28 | 13 | 41 | |
| Unmethylated | 3 | 12 | 15 | *P* = 0.0020** |
| Total | 31 | 25 | | |

*P*: Fisher's exact probability test.

transcriptional or post-transcriptional level, and was not the result of mutations in the coding region of the ER-α gene. In our previous studies, there were neither germline nor somatic mutations in the ER gene in 14 patients with ER-α-negative and PR-positive breast tumours as assessed by single-strand conformation polymorphism analysis and DNA sequencing (Iwase et al, 1996). Furthermore, we did not find a role for the loss of heterozygosity (LOH) of the ER-α gene in the lack of ER-α function in breast cancer tissues (Iwase et al, 1995). The mutation of one allele and the loss or replacement of a chromosomal segment containing the other allele were not accompanied by changes in ER-α expression. Thus, genetic alterations in the ER-α gene at the DNA level might account for only a portion of hormone independence.

The methylation of CpG islands of DNA induces a dilatation of a major groove and a kink in a minor groove at opposite sides of the double helix loop (Baylin et al, 1998). These conformation changes in chromosomes result in changes of interaction between DNA and core histone particles. There have been many reports on various genes concerning suppression of the promoter function by DNA methylation. In addition, DNA methylation of a specific gene will affect its expression (Baylin et al, 1998). Hypermethylation within the promoters of selected genes appears to be especially common in all types of human haematopoietic neoplasms, and is usually associated with inactivation of involved genes such as p15, p16 (Gonzalez-Zulueta et al, 1995) and E-cadherin (Hennig et al, 1995). The ER-α gene was found to be methylated in placental tissues, but normal breast tissues exhibited a different methylation pattern, as assessed by *Hpa*II and *Msp*I restriction enzyme digests (Falette et al, 1990). In addition, specific sites in the hormone-binding domain of the ER-α gene were observed to be differently methylated in different human breast tumour specimens. Although methylation of the ER-α gene varied among tumours, the degree of methylation did not correlate with the levels of receptor-protein expression (Falette et al, 1990; Watts et al, 1992). However, these studies used a large ER gene probe (pOR8) that corresponded to internal ER sequences. The inactivation of ER-α gene expression is associated with de novo methylation of cluster CpG sites located in and around the promoter of the gene in ER-α-negative breast tumours (Ottaviano et al, 1994; Lapidus et al, 1998) and colorectal tumours (Ahuja et al, 1997). Furthermore, unmethylation of the ER-α gene in ER-α-negative breast cancer cells treated with two inhibitors of DNA methylation, 5-azacytidine or 5-aza-2′-deoxycytidine, can reactivate ER-α gene expression (Ferguson et al, 1997). Lapidus et al (1998) reported that all samples from normal breast epithelia were unmethylated at ER-α gene CpG island using bisulphite and PCR assay. In our investigation all DNA samples extracted from normal breast tissues were unmethylated (data not shown). Thus, DNA methylation of the ER-α gene may contribute to ER-α protein expression.

We used PCR-based methylation-sensitive restriction enzymes prior to PCR amplification. However, this method has the potential of generating false positive signals (methylation present) because of inefficient enzyme digestion or overamplification in the subsequent PCR reaction. To avoid such signals, we performed the digestion of each sample and PCR amplification at least twice in independent experiments, and we confirmed the methylation status by conventional Southern hybridization. The results agreed well with those of PCR-based methylation assay. Furthermore, this region, located from 400 to 500 bp from ER-α gene start site, is the most important region of ER CpG island with respect to ER-α expression (Lapidus et al, 1998). In our data, the frequency of unmethylation at the P1 promoter region (44.6%) was higher than that at the P0 promoter region (26.8%). The correlation between ER-α expression and methylation at the P1 promoter region ($P$ = 0.0002) was higher than that at the P0 promoter region ($P$ = 0.0154). This result shows that ER-α expression might be more influenced by unmethylation at the proximal promoter region than that at the distal promoter region. Additionally, tumours with the ER-α gene hypermethylated at both promoter regions had definitely negative ER-α values. In other words, these results showed that hypermethylation at the promoter regions of the ER-α gene might be quite important for ER negativity accompanying tumour progression.

Chen et al (1998) reported that the ER-α CpG island in C4:2 cells, a subclone of T47D cells without ER-α expression, remained unmethylated. This result shows that the loss of ER-α in these specific breast cancer cells must be due to a mechanism other than methylation. However, we supposed that methylation at the distal promoter region of the ER-α gene should be examined in such cell lines, and that they might be due to a difference between clinical cases and cell lines. In our data, several cases without ER-α expression actually had unmethylation at either the P0 or P1 region of the ER gene.

In conclusion, this epigenetic change, ER gene CpG island methylation, might control ER-α expression, and might play an important role of loss in the hormone dependence in ER-α-negative recurrent tumours arising from ER-α-positive tumours. Therefore, there is a possibility that the methylation status, which can be detected from genomic DNA of the tumour, may be a good marker to determine the hormone dependency in breast tumours.

### ACKNOWLEDGEMENTS

This work was supported by a Grant-in-Aid for Scientific Research from the Ministry of Education (Project Number: 08457311, 09671241). The authors wish to thank Mrs Mariko Nishio for her excellent technical support.

### REFERENCES

Ahuja N, Mohan AL, Li Q, Stolker JM, Herman JG, Hamilton SR, Baylin SB and Issa JP (1997) Association between CpG island methylation and microsatellite instability in colorectal cancer. *Cancer Res* **57**: 3370–3374

Baylin SB, Herman JG, Graff JR, Vertino PM and Issa JP (1998) Alterations in DNA methylation: a fundamental aspect of neoplasia. *Adv Cancer Res* **72**: 141–196

Chen Z, Ko A, Yang J and Jordan VC (1998) Methylation of CpG island is not a ubiquitous mechanism for the loss of oestrogen receptor in breast cancer cells. *Br J Cancer* **77**: 181–185

© 1999 Cancer Research Campaign 

Falette NS, Fuqua SAW, Chamness GC, Cheah MS, Greene GL and McGuire WL (1990) Estrogen receptor gene methylation in human breast tumors. *Cancer Res* **50**: 3974–3978

Ferguson AT, Lapidus RG, Baylin SB and Davidson NE (1995) Demethylation of the estrogen receptor gene in estrogen receptor-negative breast cancer cells can reactivate estrogen receptor gene expression. *Cancer Res* **55**: 2279–2283

Ferguson AT, Vertino PM, Spitzner JR, Baylin SB, Muller MT and Davidson NE (1997) Role of estrogen receptor gene demethylation and DNA methyltransferase. DNA adduct formation in 5-aza-2′deoxycytidine-induced cytotoxicity in human breast cancer cells. *J Biol Chem* **272**: 32260–32266

Gonzalez-Zulueta M, Bender CM, Yang AS, Nguygen T, Beart RW, Tornout JM and Jones PA (1995) Methylation of the 5′ CpG island of the p16/CDKN2 tumor suppressor gene in normal and transformed human tissues correlates with gene silencing. *Cancer Res* **55**: 4531–4535

Grandien K, Backdahl M, Ljunggren O, Gustafsson JA and Berkenstam A (1995) Estrogen target tissue determines alternative promoter utilization of the human estrogen receptor gene in osteoblasts and tumor cell lines. *Endocrinology* **136**: 2223–2229

Hayashi S, Imai K, Suga K, Kurihara T, Higashi Y and Nakachi K (1997) Two promoters in expression of estrogen receptor messenger RNA in human breast cancer. *Carcinogenesis* **18**:(3) 459–464.

Hennig G, Behrens J, Truss M, Frisch S, Reichmann E and Birchmeier W (1995) Progression of carcinoma cells is associated with alterations in chromatin structure and factor binding at the E-cadherin promoter in vivo. *Oncogene* **11**: 475–484

Iwase H, Greenman JM, Barnes DM, Bobrow L, Hodgson S and Mathew CG (1995) Loss of heterozygosity of the estrogen receptor gene in breast cancer. *Br J Cancer* **71**: 448–450

Iwase H, Greenman JM, Barns DM, Hodgson S, Bobrow L and Mathew CG (1996) Sequence variants of the estrogen receptor (ER) gene found in breast cancer patients with ER negative and progesterone receptor positive tumors. *Cancer Lett* **108**: 179–184

Johnston SR, Saccani-Jotti G, Smith IE, Salter J, Newby J, Coppen M, Ebbs SR and Dowsett M (1995) Changes in estrogen receptor, progesterone receptor, and pS2 expression in tamoxifen-resistant human breast cancer. *Cancer Res* **55**: 3331–3338

Karnik PS, Kulkarni S, Liu XP, Budd GT and Bukowski RM (1994) Estrogen receptor mutations in tamoxifen resistant breast cancer. *Cancer Res* **53**: 349–353

Katzenellenbogen BS, Montano MM, Ekena K, Herman ME and McInerney EM (1997) Antiestrogens: mechanisms of action and resistance in breast cancer. *Breast Cancer Res Treat* **44**: 23–38.

Lapidus RG, Nass SJ, Butash KA, Parl FF, Weitzman SA, Graff JG, Herman JG and Davidson NE (1998) Mapping of ER CpG island methylation by methylation-specific polymerase chain reaction. *Cancer Res* **58**: 2515–2519.

McGuire WJ, Chamness GC and Fuqua SAW (1991) Estrogen receptor variants in clinical breast cancer. *Mol Endocrinol* **5**: 1571–1577

Ottaviano YL, Issa JP, Parl FF, Smith HS, Baylin SB and Davidson NE (1994) Methylation of the estrogen receptor gene CpG island marks loss of estrogen receptor expression in human breast cancer cells. *Cancer Res* **54**: 2552–2525

Roodi N, Bailey LR, Kao WY, Verrier CS, Yee CJ, Dupont WD and Parl FF (1995) Estrogen receptor gene analysis in estrogen receptor-positive and receptor-negative primary breast cancer. *J Natl Cancer Inst* **87**: 446–451

Watts CK, Handel ML and King RJ (1992) Oestrogen receptor gene structure and function in breast cancer. *J Steroid Biochem Mol Biol* **41**: 529–536

*© 1999 Cancer Research Campaign*
